# Dose Finding in Oncology: What is Impeding Coming of Age?

**DOI:** 10.1007/s11095-022-03263-5

**Published:** 2022-04-26

**Authors:** Kapil Mayawala, Dinesh de Alwis

**Affiliations:** Oncology Early Development, Clinical Research, Merck and Co., Inc., NJ Kenilworth, USA

## Abstract

After a drug molecule enters clinical trials, there are primarily three levers to enhance probability of success: patient selection, dose selection and choice of combination agents. Of these, dose selection remains an under-appreciated aspect in oncology drug development despite numerous peer-reviewed publications. Here, we share practical challenges faced by the biopharmaceutical industry that reduce the willingness to invest in dose finding for oncology drugs. First, randomized dose finding admittedly slows down clinical development. To reduce the size of dose finding study, trend in exposure *vs*. tumor-size analysis can be assessed, instead of a statistical test for non-inferiority between multiple doses. Second, investment in testing a lower dose when benefit-risk at the higher dose is sufficient for regulatory approval (i.e., efficacy at the higher dose is better than standard of care and safety is acceptable) is perceived as low priority. Changing regulatory landscape must be considered to optimize dose in pre-marketing setting as post-marketing changes in dose can be commercially costly. Third, the risk of exposing patients to subtherapeutic exposures with a lower dose should be assessed scientifically instead of assuming a monotonic relationship between dose and efficacy. Only the doses which are expected to be at the plateau of dose/exposure–response curve should be investigated in Phase 1b/2. Overall, changing the perceptions that have been impeding investment in dose finding in oncology requires pragmatic discourse among biopharmaceutical industry, regulatory agencies and academia. These perceptions should also not deter dose finding for recently emerging modalities, including BITEs and CART cell therapies.

Despite numerous peer-reviewed publications by biopharmaceutical industry, regulatory agencies and academia (1–6), current state of investment in dose finding in oncology by the biopharmaceutical industry is reflected by the dearth of randomized dose finding studies. Our (non-systematic) literature search for randomized dose-finding studies to support the registrational dose for an immune-oncology (IO) monoclonal antibody (mAb) over the last decade led to only one drug for which this was done – pembrolizumab (7). This is concerning considering the vast investment in IO drug development: 6,281 active clinical trials testing IO agents in 2020 (8). For antibody drug-conjugates (ADCs), another active area in oncology, randomized dose finding is also uncommon with only 2 examples: trastuzumab deruxtecan (9) and belantamab mafodotin (10). Given the current state of dose finding in oncology, progress depends more on recognition of pragmatic challenges to dose finding than on investment in developing novel methodologies. Here, we share our opinion on key challenges in the biopharmaceutical industry that reduce the willingness to invest in dose finding for oncology drugs (Fig. 1).

**Fig. 1:**
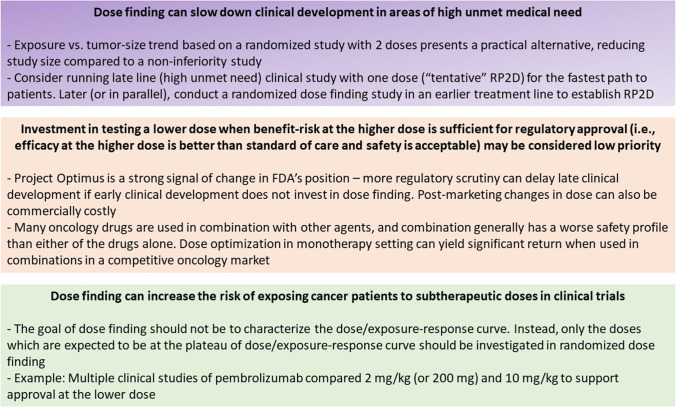
Practical challenges faced by the biopharmaceutical industry and considerations that should be part of modern clinical development in oncology (independent of modality)

## Randomized Dose Finding can Slow Down Clinical Development in Areas of High Unmet Medical Need

Admittedly, exploring more doses requires additional patients which slows down development timelines. This challenge can be addressed to some extent in following ways:

1. In addition to dose *vs*. overall response rate (ORR) assessment using data from > 1 dose level to establish optimal dose, exposure *vs*. tumor-size trend can be investigated to assess the probability of higher efficacy at a higher dose (11). Such an analysis focused on characterizing dose/exposure–response trends, instead of a statistical test for non-inferiority between multiple doses, has the potential to inform optimal dose with fewer patients (4, 12). A trend between exposure *vs*. tumor-size may also add to the confidence in proof of concept.
2. Initial clinical study, if in a late treatment line with high unmet need and small patient population, may be run at one dose for the fastest path to approval and patients. However, this dose should not be assumed to be the maximum tolerated dose (MTD) or maximum administered dose (MAD). Instead, it should be selected by integrating all available data on pharmacokinetics (PK), pharmacodynamics (PD)/biomarkers, efficacy and safety together with understanding of mechanism of action. Later (or in parallel with the initial study in a late line setting), a randomized dose finding study can be conducted in an earlier treatment line to establish optimal dose for maximizing benefit-risk in a wider patient population.

## Investment in Testing a Lower Dose When Benefit-Risk at the Higher Dose is Sufficient for Regulatory Approval (i.e., Efficacy at The Higher Dose is Better Than Standard of Care and Safety is Acceptable) may be Considered Low Priority

Changing regulatory landscape must be considered to inform what is sufficient for approval instead of relying on the general precedent in oncology. Recently, a multiple myeloma small molecule drug melphalan flufenamide was pulled off the market following accelerated approval due to poor efficacy in the confirmatory trial. There is a prospect that a dose finding trial may need to be conducted before the Phase 3 study (12). As another example, the Food and Drug Administration (FDA) issued a post-marketing requirement (PMR) for Amgen to compare sotorasib’s approved 960 mg dose with a fourfold lower 240 mg dose (13). While several PMRs for dose optimization have been issued in the past (1, 14), a few points about sotorasib’s PMR are relevant to point to changing regulatory landscape (13).

1. Sotorasib’s accelerated approval was based on an ORR of 36% (95% CI: 28–45%) and median duration of response of 10.0 months (95% CI, 6.9–not estimable) for patients with advanced NSCLC with KRAS G12C mutation who had progressed after at least one line of therapy. Willingness to invest in dose finding by the biopharmaceutical industry is even lower for drugs such as sotorasib where a remarkable early efficacy signal ensures path to regulatory approval.
2. FDA review did not find conclusive dose/exposure–response relationship for safety, albeit a trend towards higher Grade 3 + treatment emergent AEs and higher Grade 3 + gastrointestinal disorders with higher exposure was reported. Even though safety was acceptable at 960 mg, there remained a possibility of improvement at a lower dose. Notably, such a possibility exists for many targeted drugs, particularly small molecules, when benefit-risk is only assessed at one dose.

Sotorasib’s PMR was not primarily driven by safety considerations, instead by the totality of PK, efficacy and safety data, along with potential to improve pill burden for patient convenience. If the 240 mg dose is found to provide better (or similar) benefit-risk than 960 mg, the commercial implications of fourfold reduction in dose can be significant as drugs are mostly priced based on dosage amounts.

A recent FDA guidance, Expansion Cohorts: Use in First-in-Human Clinical Trials to Expedite Development of Oncology Drugs and Biologics, emphasizes more investment in dose finding (15). The FDA is more actively looking for ways to engage sponsors on dose finding in the pre-marketing setting, e.g., Oncology Center of Excellence’s recent initiative Project Optimus (1). Importantly, Project Optimus should not be perceived merely as added regulatory requirement, instead as an initiative to change the mindset around dose finding in oncology to eventually improve benefit-risk for cancer patients and to bring different drug developers at the same level. Project Optimus places emphasis on performing dose finding studies early and efficiently during clinical development. To balance speed and the need for right dose, relationship between drug developers and FDA as partners is essential to develop pragmatic dose selection strategies based on totality of scientific evidence.

Many oncology drugs are used in combination with other agents, and a combination generally has a worse safety profile than either of the drugs alone. So even for a drug with acceptable benefit-risk profile as monotherapy, dose optimization to improve its safety profile in monotherapy setting can yield significant return when used in combinations. This can be part of the differentiation strategy in a highly competitive oncology landscape with multiple companies working on the same targets in some cases.

## Dose Finding can Increase the Risk of Exposing Cancer Patients to Subtherapeutic Doses in Clinical Trials

Instead of assuming a monotonic relationship between dose and efficacy, the risk of exposing patients to subtherapeutic exposures with a lower dose should be assessed scientifically, informed by the body of preclinical and clinical data. To minimize the chance of exposing cancer patients to subtherapeutic doses in Phase 1b/2, the goal of dose finding should not be to characterize the dose/exposure–response curve. Instead, only the doses which are expected to be at the plateau of dose/exposure–response curve should be investigated in Phase 1b/2. As an example, 200 mg Q3W (fixed dose equivalent of 2 mg/kg) was defined as the tentative recommended phase 2 dose (RP2D) for pembrolizumab as it was predicted to achieve target saturation based on modeling and simulation using early clinical data from Phase 1 (16), thereby maximizing downstream pharmacology and chances of efficacy in cancer patients (17). To confirm that tentative RP2D maximizes benefit-risk, randomized dose comparisons were performed to compare tentative RP2D and maximum administrated dose (10 mg/kg), which was fivefold higher, to establish 200 mg as the optimal dose (7, 11).

The importance of randomized studies is demonstrated by early clinical data in the pembrolizumab program. For ipilimumab-naïve melanoma patients, ORRs (assessed using immune-related response criteria) of 14, 33, and 56% were reported for 2 mg/kg every 3 weeks (Q3W), 10 mg/kg every 3 weeks (Q3W), and 10 mg/kg every 2 weeks (Q2W), respectively (18). Non-randomized comparisons based on early clinical data showed a dose–response relationship suggesting maximal efficacy with 10 mg/kg Q2W. Later, randomized dose comparison studies showed that there was no efficacy advantage in dosing above 200 mg (fixed dose equivalent of 2 mg/kg) Q3W (7, 11). Clinical data from the dose finding studies also later enabled a modeling and simulation based approval of an every 6 weeks dosing regimen for pembrolizumab (19) – further increasing returns, in terms of patient convenience, on investment in dose finding, particularly during the Covid pandemic.

Admittedly, lack of validated biomarkers, especially for drugs with novel mechanisms of action, is a challenge for the selection of tentative RP2D. Tumor size, both at patient level and individual lesion level, can be used as an effective marker of a drug’s activity (4). In addition, target engagement (TE) is a key determinant of pharmacology by antagonizing IO mAbs. Pembrolizumab is an example of how tentative RP2D can be selected based on TE, where a physiology-based pharmacokinetic model was used to predict dose required for target saturation in blood and in tumor microenvironment (16, 17).

TE can also be used to inform dose selection of ADCs – a rapidly emerging area in oncology. Probability of success with an ADC depends on several factors including antigen selection and payload potency as well as drug properties such as affinity and linker stability (20). Mechanism of action of ADCs involves intracellular payload delivery by binding to membrane antigen followed by internalization. Therefore, a quantitative characterization of TE in terms of area under the curve (AUC) of TE can be a useful measure of total payload delivery over time (additional payload delivery can also occur by bystander effect). Such a measure can also consider the impact of any downregulation of antigen over time (20). Achieving optimal AUC of TE can be enabled by optimizing the dosing regimen of ADCs, which can have shorter half-lives than naked mAbs (20). With similar AUC of PK exposure, a lower dose given more frequently can achieve higher AUC of TE than a higher dose given less frequently, potentially improving therapeutic index for ADCs in some cases. For example, Phase 1 study of telisotuzumab vedotin initially studied a Q3W regimen and established 2.7 mg/kg Q3W as RP2D (21). Later, a more frequent regimen of Q2W was also studied and 1.9 mg/kg Q2W was established as an alternate RP2D (22). Recently, telisotuzumab vedotin was granted breakthrough designation based on a Phase 2 trial (Luminosity) which used 1.9 mg/kg Q2W. The impressive clinical data was likely contributed by right patient selection (cMET + NSCLC) as well as using the right dosing regimen (Q2W). The importance of AUC of TE is also suggested by switching enfortumab vedotin’s dosing from a less frequent regimen (Q3W) tested in FIH study to the approval a more frequent regimen (once weekly for the first 3 weeks of every 4 weeks), informed by enfortumab vedotin’s relatively short half-life of ~ 3.4 days (23).

To minimize the chances of subtherapeutic exposures, TE profile should particularly be considered for ADCs using highly toxic payloads such as pyrrolobenzodiazepine (PBD) (24). For example, rovalpituzumab tesirine, a PBD-based ADC, was administered for only two doses (median) of 0.3 mg/kg six weeks apart due to safety. Many of the adverse events were similar to what have been observed with other PBD-based ADCs suggesting payload-mediated toxicities contributed to safety profile (24). As a result of the dosing scheme together with poor penetration of mAbs into solid tumors, it may be expected that rovalpituzumab tesirine would have led to a poor AUC of TE on tumor cells (25, 26). In Phase 3, rovalpituzumab tesirine was inferior to standard of care with hazard ratios of 1.51 (95% CI: 1.22–1.87) for progression-free survival and 1.46 (95% CI: 1.17–1.82) for overall survival (27).

Finally, as more complex modalities, such as bi-specific T cell engagers (BiTEs) and chimeric antigen receptor T (CART) cell therapies, are emerging in pipelines across the biopharmaceutical industry, selecting the right dose based on clinical pharmacology principles, e.g., assessment of patient factors influencing exposure–response, remains important during clinical development (28). In addition to the paucity of biomarkers that correlate with clinical response, a key challenge in dose selection for BITEs (and more generally for agonist mAbs) is that the optimal TE is generally poorly understood, unlike for antagonist mAbs where TE ≥ 90% is generally used as a therapeutic goal. A review by the FDA showed that 67% of BITEs reached MTDs at TE < 10% (29). Therefore, dose optimization for BITEs can likely be driven by the balance of early efficacy markers, e.g., tumor-size change and safety, e.g., cytokine release syndrome (CRS). Notably, dose titration has been utilized to improve therapeutic index of BITEs – an approach which is expected to reduce CRS (30).

CRS has also been an issue with CART cell therapies (31). Six autologous CART cell therapies have been approved by the FDA: 4 targeting CD19 and 2 targeting BCMA (32, 33). Exposure–response analyses of five of these CART cell therapies show higher exposure (AUC0-28 days) in responders compared to non-responders—as much as ~ eightfold higher for brexucabtagene autoleuce (32). Even though the exposure with CART cell therapies depends not only on dose but also on patient factors influencing cellular kinetics, dose optimization is still an area where further investigation is needed, particularly considering complexity and cost of the treatment. A randomized dose optimization study also suggested more complete responses were achieved with a higher dose of anti-CD19 CART cells (34). For allogeneic CART cell therapies with > 1 drug administration, dose optimization should be considered an important part of clinical development plan to maximize benefit-risk for patients.

In summary, changing the perceptions that have been impeding investment in dose finding in oncology require more pragmatic discussions. Additional costs and timeline impact should be balanced with the reward of improving benefit-risk for patients, which will also lead to economic benefits in a competitive oncology landscape. Regulatory agencies can also play a key role in emphasizing the importance of developing oncology drugs at the right dose. Importantly, partnership between drug developers and regulators as part of Project Optimus will be important to change the mindset of drug developers in oncology.
