# Correction to: Dose Finding in Oncology: What is Impeding Coming of Age?

**DOI:** 10.1007/s11095-022-03300-3

**Published:** 2022-06-02

**Authors:** Kapil Mayawala, Dinesh de Alwis

**Affiliations:** Clinical Research, Oncology Early Development, Merck and Co., Inc., Kenilworth, NJ USA

**Correction to** **:** **Pharmaceutical Research**

https://doi.org/10.1007/s11095-022-03263-5

The article “Dose Finding in Oncology: What is Impeding Coming of Age?,” written by Kapil Mayawala and Dinesh de Alwis, was originally published electronically on the publisher’s internet portal on 26 April 2022 without open access. With the author(s)’ decision to opt for Open Choice the copyright of the article changed on 20 May 2022 to © Merck & Co., Inc., Rahway, NJ, USA and its affiliates 2022 and the article is forthwith distributed under the terms of the Creative Commons Attribution 4.0 International License, which permits use, sharing, adaptation, distribution and reproduction in any medium or format, as long as you give appropriate credit to the original author(s) and the source, provide a link to the Creative Commons licence, and indicate if changes were made. The images or other third party material in this article are included in the article’s Creative Commons licence, unless indicated otherwise in a credit line to the material. If material is not included in the article’s Creative Commons licence and your intended use is not permitted by statutory regulation or exceeds the permitted use, you will need to obtain permission directly from the copyright holder. To view a copy of this licence, visit http://creativecommons.org/licenses/by/4.0.

